# Eye Bank Records on Pediatric Keratoplasty

**DOI:** 10.18502/jovr.v17i3.11569

**Published:** 2022-08-15

**Authors:** Seyed Mohamadmehdi Moshtaghion, Mohammad Abolhosseini, Bahareh Kheiri, Mohammad Ali Javadi, Leila Ziaee Ardakani, Mozhgan Rezaei Kanavi

**Affiliations:** ^1^Ocular Tissue Engineering Research Center, Research Institute for Ophthalmology and Vision Science, Shahid Beheshti University of Medical Sciences, Tehran, Iran; ^2^Ophthalmic Research Center, Research Institute for Ophthalmology and Vision Science, Shahid Beheshti University of Medical Sciences, Tehran, Iran; ^3^Central Eye Bank of Iran, Tehran, Iran; ^4^School of Medicine, Shahid Beheshti University of Medical Sciences, Tehran, Iran

**Keywords:** Deep Anterior Lamellar Keratoplasty, Descemet Membrane Stripping Automated Endothelial Keratoplasty, Keratoconus, Pediatric Keratoplasty, Penetrating Keratoplasty

## Abstract

**Purpose:**

To report eye bank records for pediatric keratoplasty in Iran between 2006 and 2019.

**Methods:**

In a retrospective study, all electronic records of the Central Eye Bank of Iran for pediatric keratoplasty between April 2006 and March 2019 were analyzed in terms of indications for keratoplasty, surgical techniques, their corresponding trends, and post-transplantation graft clarity.

**Results:**

Our database included 2178 eyes from 2050 pediatric cases. The leading indications for keratoplasty included acquired nontraumatic diseases (75.8%), congenital abnormalities (12.7%), corneal regraft (8.3%), and acquired traumatic diseases (3.2%). Keratoconus was the most common acquired nontraumatic cause (58%) and more common in the age group > 12 years than those ≤ 12 years (*P* < 0.001). Congenital corneal abnormalities and regrafts were more common in the age group ≤ 12 years (both *P* < 0.001). The most common surgical technique was penetrating keratoplasty (PKP, 90.9%) followed by deep anterior lamellar keratoplasty (DALK, 7.3%), Descemet stripping automated endothelial keratoplasty (DSAEK, 1.1%), anterior lamellar keratoplasty (0.5%), and keratolimbal allograft transplantation (0.2%). DSAEK was more common in the age group ≤ 12 years (*P* = 0.002), which, unlike PKP and DALK, showed a significant ascending trend over the 14-year period (*P* = 0.018). Post-transplantation graft clarity was 96.8%.

**Conclusion:**

Keratoconus was the leading indication for pediatric keratoplasty in Iran. Although PKP was the predominant keratoplasty procedure for the treatment of pediatric corneal disorders, it showed a significant descending trend over the 14 years.

##  INTRODUCTION

One of the major pediatric health problems especially in developing countries is corneal blindness.^[1]^ The etiology of corneal blindness varies depending on the differences in the race, region, hygienic conditions, and economical status. For instance, unlike developed countries in which corneal congenital disorders are the leading indication for pediatric keratoplasty,^[2,3,4,5,6]^ infectious keratitis and corneal traumatic injuries have been the major indications in the developing countries.^[7,8,9]^

Corneal transplantation is the ultimate treatment of corneal blindness in children.^[10]^ Given that it is a challenging procedure, due to its preoperative, intraoperative, and postoperative considerations,^[1]^ it is not performed very routinely.^[11]^ However, with the adoption of partial thickness microsurgical techniques, the numbers as well as the success rates of pediatric keratoplasty have increased over the last decade;^[2,4]^ the surgical techniques in cases with congenital hereditary endothelial dystrophy (CHED) and anterior stromal disorders have transitioned from full thickness to lamellar keratoplasty techniques.^[6,9]^ Nevertheless, penetrating keratoplasty (PKP) still accounts for 90% of all pediatric keratoplasties performed in 95 countries.^[10]^ Currently, pediatric keratoplasty is predominantly performed in the university-based ophthalmic centers in Iran. Tissue requirements for corneal transplantation are mainly provided by the Central Eye Bank of Iran located in the capital, Tehran. In this study, we intend to investigate the records of the Central Eye Bank of Iran in terms of indications for keratoplasty and their corresponding trends, surgical techniques and their corresponding evolving trends, as well as postoperative graft clarity between 2006 and 2019.

##  METHODS

### Study Design

The current study was approved by the Ethics Committee of the Research Institute for Ophthalmology and Vision Science affiliated to Shahid Beheshti University of Medical Sciences, Tehran, Iran (IR.SBMU.ORC.REC.1384.8).

### Population and Measurements

All eye bank records of pediatric keratoplasty cases ( ≤ 18 years old) performed between April 2006 and March 2019 throughout the country were compiled from the Central Eye Bank of Iran. The patients' data were reviewed in terms of demographic data, indications for keratoplasty and their corresponding trends, surgical techniques and their corresponding trends, as well as post-transplantation graft clarity reported to the Central Eye Bank of Iran. All variables were also analyzed in two subgroups of ≤ 12 and > 12 years of age.

### Indications for Keratoplasty

Indications for keratoplasty, according to the previous studies, were categorized into four groups:^[12,13]^ congenital corneal abnormalities (corneal dystrophies, congenital corneal opacities), acquired nontraumatic diseases (keratoconus, corneal degenerations, aphakic/pseudophakic bullous keratopathies, corneal infections, and non-specified corneal opacities), acquired traumatic diseases (mechanical injuries, chemical or thermal injuries), and regraft. The regraft group was also investigated for the cause of graft failure and the original indications for keratoplasty.

### Surgical Techniques

PKP, Descemet stripping automated endothelial keratoplasty (DSAEK), deep anterior lamellar keratoplasty (DALK), anterior lamellar keratoplasty (ALK), and keratolimbal allograft transplantation (KLAL) were surgical techniques implemented for pediatric keratoplasty in our survey.

### Statistical Analyses

Statistical analyses were performed using the SPSS software (version 25; SPSS Inc., Chicago, IL, USA). Data were presented as mean ± standard deviation. Linear regression analyses were applied to analyze the trend of indications and surgical techniques over time. Considering statistical values such as goodness of fit and R-square, the SPSS chose the best model to determine the regression process. *P*-values < 0.05 were considered statistically significant.

##  RESULTS

### Patients

Our database included 2178 eye bank records from 2050 pediatric cases that had undergone pediatric keratoplasty between 2006 and 2019. The patients' age ranged from 1 month to 18 years with the mean age of 11.2 ± 4.5 years at the time of surgery, where 61% were male. The 128 eye bank records corresponded to the patients in whom either the fellow eyes were transplanted (70 eyes from 70 patients) or the transplanted eyes underwent regraft (58 eyes from 56 patients). The annual rates of pediatric keratoplasty revealed a significant decline of trend over the 14-year period (*P* < 0.001). Out of the 2178 eye bank records, 1061 (48.7%) cases were ≤ 12 years old and the remaining 1117 (51.3%) were > 12 years of age.

### Indications for Corneal Transplantation

The leading indications for pediatric keratoplasty, in order of descending frequency, were acquired nontraumatic diseases (1651 eyes, 75.8%), congenital corneal abnormalities (277 eyes, 12.7%), regraft (181 eyes, 8.3%), and acquired traumatic diseases (69, 3.2%) [Table 1].

### Acquired nontraumatic diseases 

**Figure 1:**
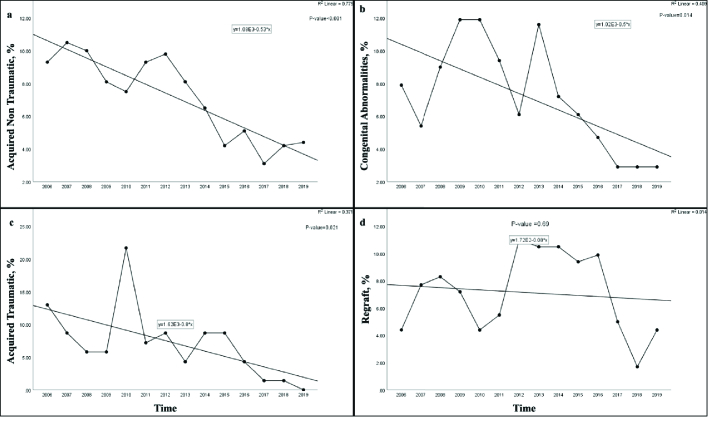
Trends of indications for pediatric keratoplasty between 2006 and 2019. Note the significant falling trend for acquired non-traumatic diseases (a; *P* < 0.001); congenital abnormalities (b; *P* = 0.014), and acquired traumatic diseases (c; *P* = 0.021). No significant change of trend is observed for the regraft over the 14-year period (d; *P* = 0.69).

The acquired nontraumatic diseases indicated a significant downward trend (*P* < 0.001) over the 14 years [Figure 1a] and were more frequent in the older ( > 12 years) than the younger age ( ≤ 12 years) group (*P* < 0.001) [Table 1]. The most common acquired nontraumatic disease was keratoconus (956, 58%) followed by aphakic/pseudophakic bullous keratopathies (258, 15.6%), non-specified corneal opacities (244, 14.8%), corneal infections (179, 10.8%), and corneal degenerations (12, 0.7%) [Table 1]. Active corneal infections accounted for 84.4% (151 eyes) of the eyes that were categorized as corneal infection.

**Figure 2:**
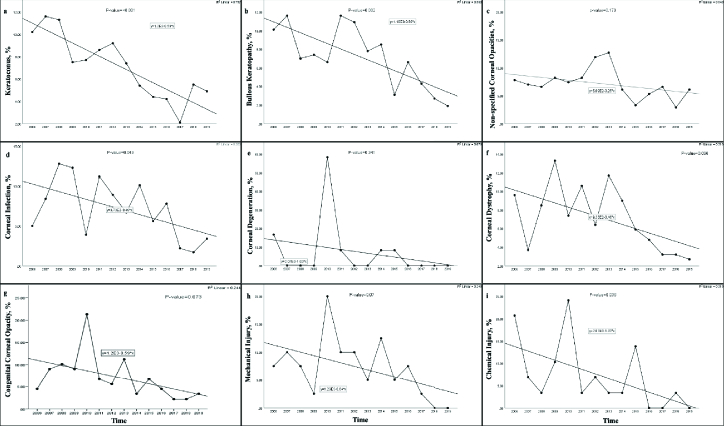
Trends of the indications for pediatric keratoplasty in each main category in Iran between 2006 and 2019. Note the significant declining trend for keratoconus (a; *P* < 0.001); bullous keratopathies (b; *P* = 0.002); corneal infections (d; *P* = 0.043); corneal dystrophies (f; *P* = 0.036); and chemical injuries (i; *P* = 0.036). No significant change of trend is noted for non-specified corneal opacities (c; *P* = 0.173), corneal degenerations (e; *P* = 0.341), congenital corneal opacities (g; *P* = 0.073), and mechanical injuries (h; *P* = 0.07) over the 14-year period.

As illustrated in Figure 2a–2e, a significant descending trend was noted in the keratoconus (*P* < 0.001), aphakic/pseudophakic bullous keratopathies (*P* = 0.002), and corneal infections (*P* = 0.043) over the 14-year period. Unlike keratoconus which was more common in the elder age group (*P* < 0.001), aphakic/pseudophakic bullous keratopathies, non-specified corneal opacities, and corneal infections were more common in the younger age group (all *P* < 0.001). In the age group ≤ 12 years, the rate of keratoconus (235 eyes, 22.1%) was even higher than the rate of congenital corneal abnormalities (196 eyes, 18.5%) (*P* = 0.035).

### Congenital corneal abnormalities

Congenital corneal abnormalities showed a significant diminishing trend (*P* = 0.014) over the 14-year period [Figure 1b] and were higher in the younger than the older age group (*P* < 0.001) [Table 1]. The most common congenital corneal abnormality was corneal dystrophy (188, 67.9%), followed by congenital corneal opacities (89, 32.1%). Both corneal dystrophies and congenital corneal opacities were more common in the younger age group (*P* < 0.001) [Table 1]. Unlike the meaningful descending trend of corneal dystrophies (*P* = 0.036), the declining trend of congenital corneal opacities over the 14 years was not statistically significant (*P* = 0.073) [Figure 2f–2g].

Out of the 188 eyes that underwent corneal transplantation for corneal dystrophies, CHED was present in 77.7% (146), followed by macular corneal dystrophy (*n* = 24, 12.7%), granular corneal dystrophy (*n* = 13, 6.9%), lattice corneal dystrophy (*n* = 3, 1.6%), and Reis-Buckler corneal dystrophy (*n* = 2, 1.1%) [Table 1]. Congenital corneal opacities (89 eyes) in our survey included limbal dermoids (41, 46.1%), Peter's anomaly (25, 28.1%), posterior keratoconus (10, 11.2%), sclerocornea (9, 10.1%), and congenital glaucoma (4, 4.5%).

### Regraft 

Regraft did not indicate any significant change of trend (*P* = 0.69) over the 14-year period [Figure 1d] and was more frequently observed in the younger than the older age group (*P* < 0.001) [Table 1]. The causes of graft failure in 181 regraft cases were chronic endothelial graft rejection/dysfunction of the allograft in 158 (87.3%) and primary graft failure in 23 (12.7%) eyes. According to the database of the Central Eye Bank of Iran, the original diagnoses were specified only in 58 eyes of 56 pediatric cases, where the five leading diagnoses were congenital corneal abnormalities (18 eyes, 31%), keratoconus (13 eyes, 22.4%), non-specified corneal opacities (13 eyes, 22.4%), aphakic/pseudophakic bullous keratopathies (6 eyes, 8.6%), and corneal infections (4 eyes, 6.9%). The leading indications in the category of primary graft failure (23 eyes) were keratoconus in six, congenital corneal abnormalities in five, and corneal infections in four eyes.

### Acquired traumatic diseases

Acquired traumatic diseases revealed a significant downward trend (*P* = 0.021) over the 14 years [Figure 1c] and were more common in the younger age group (*P* = 0.012) [Table 1]. The most common acquired traumatic disease was mechanical injuries (40, 58%), followed by chemical injuries (29, 42%). Unlike mechanical injuries which were more common in the younger age group (*P* < 0.001) [Table 1], chemical injuries were not different between the two age groups (*P* = 0.746). Unlike the borderline change of trend in the mechanical injuries (*P* = 0.07), chemical injuries indicated a significant falling trend (*P* = 0.036) over the 14-year period [Figure 2h & 2i].

**Table 1:** Indications for pediatric keratoplasty in Iran between 2006 and 2019 and the corresponding distribution in two age groups of ≤ 12 and > 12 years.


**Distribution of the indications for pediatric keratoplasty of each age group**				
**Preoperative diagnosis**	**Total**	**Age group (yr)**		***P*** **-value **
	≤ 12	12 <	
Mean age	11.2 ± 4.5	7.5 ± 3.8	14.5 ± 1.6	< 0.001
**Acquired non-traumatic**	**1651 (75.8%)**	**703 (66.3%)**	**948 (84.9%)**	** < 0.001**
Keratoconus	958 (58.0%)	235 (33.4%)	723 (76.3%)	< 0.001
Aphakic/Pseudophakic bullous keratopathy	258 (15.6%)	171 (24.3%)	87 (9.2%)	< 0.001
Non-specified corneal opacities	244 (14.8%)	161 (22.9%)	83 (8.8%)	< 0.001
Corneal infections	179 (10.8%)	128 (18.2%)	51 (5.4%)	< 0.001
* Viral *	27 (15.1%)	15 (11.7%)	12 (23.5%)	0.057
* Bacterial *	61 (34.1%)	42 (32.8%)	19 (37.3%)	0.573
* Fungal *	13 (7.3%)	9 (7%)	4 (7.8%)	0.831
* Amebic *	0 (0%)	0 (0%)	0 (0%)	N/A
* Unknown *	78 (43.6%)	62 (48.4%)	16 (31.4%)	0.038
Corneal degenerations	12 (0.7%)	8 (1.1%)	4 (0.4%)	0.223
**Congenital corneal abnormalities**	**277 (12.7%)**	**196 (18.5%)**	**81 (7.3%)**	** < 0.001**
Corneal dystrophies	188 (67.9%)	129 (65.8%)	59 (72.8%)	< 0.001
* CHED *	146 (77.7%)	117 (90.7%)	29 (49.2%)	< 0.001
* MCD *	24 (12.7%)	5 (3.9%)	19 (32.2%)	< 0.001
* GCD *	13 (6.9%)	3 (2.3%)	10 (16.9%)	< 0.001
* LCD *	3 (1.6%)	3 (2.3%)	0 (0%)	0.320
* Reis-Buckler corneal dystrophy *	2 (1.1%)	1 (0.8%)	1 (1.7%)	0.627
Congenital corneal opacity	89 (32.1%)	67 (34.2%)	22 (27.2%)	< 0.001
*Limbal dermoids*	41 (46.1%)	35 (52.2%)	6 (27.3%)	0.023
*Peter's anomaly*	25 (28.1%)	14 (20.9%)	11 (50%)	0.005
*posterior keratoconus*	10 (11.2%)	6 (9%)	4 (18.2%)	0.121
*Sclerocornea*	9 (10.1%)	9 (13.4%)	0 (0%)	0.128
*congenital glaucoma*	4 (4.5%)	3 (4.5%)	1 (4.5%)	0.494
**Acquired traumatic**	**69 (3.2%)**	**44 (4.1%)**	**25 (2.2%)**	**0.012**
Mechanical injuries	40 (58.0%)	29 (65.9%)	11 (44.0%)	0.003
Chemical injuries	29(42.0%)	15 (34.1%)	14 (56.0%)	0.746
**Regraft**	**181 (8.3%)**	**118 (11.1%)**	**63 (5.6%)**	** < 0.001**
Total	2178 (100.0%)	1061 (100.0%)	1117 (100.0%)	0.09
	
	
CHED, congenital hereditary endothelial dystrophy; MCD, macular corneal dystrophy; GCD, granular corneal dystrophy; LCD, lattice corneal dystrophy				

**Table 2:** Number of surgical techniques used for pediatric keratoplasty between 2006 and 2019, and the corresponding distribution in two age groups of ≤ 12 and > 12 years.


**Surgical techniques**	**Total**	**Age group (yr)**			***P*** **-value**
	** ≤ 12**	** > 12**	
PKP	1981 (90.9%)	998 (94.1%)	983 (88.0%)	0.634
DALK	159 (7.3%)	38 (3.6%)	121 (10.8%)	< 0.001
DSAEK	24 (1.1%)	19 (1.8%)	5 (0.4%)	0.002
ALK	10 (0.5%)	3 (0.3%)	7 (0.6%)	0.082
KLAL	4 (0.2%)	3 (0.3%)	1 (0.1%)	0.178
			
**Sum**	**2178 (100%)**	**1061 (48.7%)**	**1117 (51.3%)**	**0.777**
	
	
PKP, penetrating keratoplasty; DALK, deep anterior lamellar keratoplasty; DSAEK, Descemet stripping automated endothelial keratoplasty; ALK, anterior lamellar keratoplasty; KLAL, keratolimbal allograft					

**Table 3:** Publications on pediatric keratoplasty since 1968, in the developed and developing countries including the present study.


**Country/Year**		**Number of eyes/ Patients**	**Acquired non-traumatic**	**Keratoconus**	**Aphakic/ Pseudophakic bullous keratopathy**	**Non-specified corneal opacities**	**Corneal infections**	**Corneal degenerations**	**Congenital corneal abnormalities**	**Corneal dystrophies/ CHED/ Non-CHED**	**Congenital corneal opacity**	**Acquired traumatic**	**Regraft**	**Total Keratoplasties**
Developing Countries	Iran/2006-2019	2178/2050	1651 (75.8%)	958 (44%)	258 (11.9%)	244 (11.2%)	179 (8.2%)	12 (0.6%)	277 (12.7%)	188/146/42(8.6%/ 6.7%/1.9%)	0	69 (3.2%)	181 (8.3%)	2178 (100%)
	Brazil/2019	51/43	9 (17.7%)	N/S	N/S	N/S	9 (17.7%)	N/S	37 (72.6%)	8/8/N/S(15.7%/8%)	29 (56.9%)	5 (9.8%)	N/S	51 (100%)
	Eastern China/2008-2017	1059/1026	175 (16.5%)	118 (11.1%)	5 (0.5%)	24 (2.3%)	26 (2.5%)	2 (0.2%)	790 (74.6%)	8/ N/S / N/S (0.8%)	782 (73.8%)	38 (3.6%)	56 (5.3%)	1059 (100%)
	Malaysia/2008-2017	16/14	10 (62.5%)	N/S	N/S	1 (6.3%)	9 (56.3%)	N/S	3 (18.8%)	N/S	3 (18.8%)	2 (12.5%)	1 (6.3%)	16 (100%)
	Mexico/1995-2011	574/(N/S)	461 (80.3%)	319 (55.6%)	35 (6.1%)	19 (3.3%)	88 (15.3%)	N/S	70 (12.2%)	N/S	70 (12.2%)	43 (7.5%)	N/S	574 (100%)
	India/2007-2011	66/66	33 (50%)	N/S	N/S	N/S	22 (33.3%)	11 (16.7%)	24 (36.4%)	18/6/12 (27.3%/9.1%/18.2%)	6 (9.1%)	5 (7.6%)	4 (6.1%)	66 (100%)
	Tunisia/2003-2008	16/15	8 (50%)	5 (31.3%)	N/S	N/S	3 (18.8%)	N/S	2 (12.5%)	1/ N/S / N/S (6.3%)	1 (6.3%)	6 (37.5%)	N/S	16 (100%)
	China (Shanghai)/2003-2007	156/149	71 (45.5%)	17 (10.9%)	N/S	8 (5.1%)	46 (29.5%)	N/S	37 (23.7%)	7/7/ N/S (4.5%/4.5%)	30 (19.2%)	48 (30.8%)	N/S	156 (100%)
	North China/1994-2005	410/371	148 (36.1%)	37 (9%)	N/S	11 (2.7%)	93 (22.7%)	7 (1.7%)	112 (27.3%)	29/ N/S / N/S (7.1%)	83 (20.2%)	150 (36.6%)	N/S	410 (100%)
	Saudi Arabia/1990-2003	165/134	18 (10.9%)	N/S	N/S	N/S	18 (10.9%)	N/S	130 (78.8%)	35/35/ N/S (21.2%)	95 (57.6%)	17 (10.3%)	N/S	165 (100%)
	India/1998-2004	168/154	89 (53%)	N/S	2 (1.2%)	N/S	73 (43.5%)	14 (8.3%)	57 (33.9%)	14/ N/S / N/S (8.3%)	43 (25.6%)	22 (13.1%)	N/S	168 (100%)
	India/1988-1995	162/140	85 (52.5%)	N/S	N/S	31 (19.1%)	54 (33.3%)	N/S	47 (29%)	20/20/ N/S (12.4%/12.4%)	27 (16.7%)	22 (13.6%)	8 (4.9%)	162 (100%)
Developed Countries	USA/2005-2017	2620/(N/S)	1931 (73.7%)	883 (33.7%)	118 (4.5%)	836 (31.9%)	94 (3.6%)	N/S	419 (16%)	262/58/204 (10%/2.2%/7.8%)	157 (%6)	79 (3%)	191 (7.3%)	2620 (100%)
	Finland/1968-2011	39/(N/S)	16 (41%)	7 (18%)	N/S	7 (18%)	1 (2.6%)	1 (2.6%)	12 (30.8%)	4/ N/S / N/S (10.3%)	8 (20.5%)	11 (28.2%)	N/S	39 (100%)
	Italy/2010-2013	54/43	30 (55.6%)	20 (37%)	2 (3.7%)	6 (11.1%)	2 (3.7%)	N/S	13 (24.1%)	N/S	13 (24.1%)	6 (11.1%)	5 (9.3%)	54 (100%)
	Singapore/1991-2011	105/105	58 (45.7%)	15 (11.8%)	5 (3.9%)	26 (20.5%)	12 (9.5%)	N/S	45 (35.4%)	2/1/1 (1.6%/0.8%/0.8%)	43 (33.9%)	2 (1.6%)	22 (17.3%)	127 (100%)
	Denmark/1968-2008	63/60	38 (52.1%)	12 (16.4%)	N/S	5 (6.8%)	21 (28.8%)	N/S	13 (17.8%)	N/S	13 (17.8%)	9 (12.3%)	13 (17.8%)	73 (100%)
	USA (California)/1991-2006	60/47	13 (12.3%)	2 (1.9%)	N/S	N/S	11 (10.4%)	N/S	37 (34.9%)	7/7/ N/S (6.6%/6.6%)	30 (28.3%)	10 (9.4%)	46 (43.4%)	106 (100%)
	New Zealand/1991-2003	58/52	43 (74.1%)	39 (67.2%)	N/S	N/S	4 (6.9%)	N/S	9 (15.5%)	7/2/5 (12.1%/3.5%/8.6%)	2 (3.5%)	6 (10.3%)	N/S	58 (100%)
	Australia/1984-2002	19/16	0 (0%)	N/S	N/S	N/S	N/S	N/S	8 (42.1%)	2/1/1 (10.5%/5.3%/5.3%)	6 (31.6%)	11 (57.9%)	N/S	19 (100%)
	
	
white<bcol>15</ecol>CHED, congenital hereditary endothelial dystrophy

**Figure 3:**
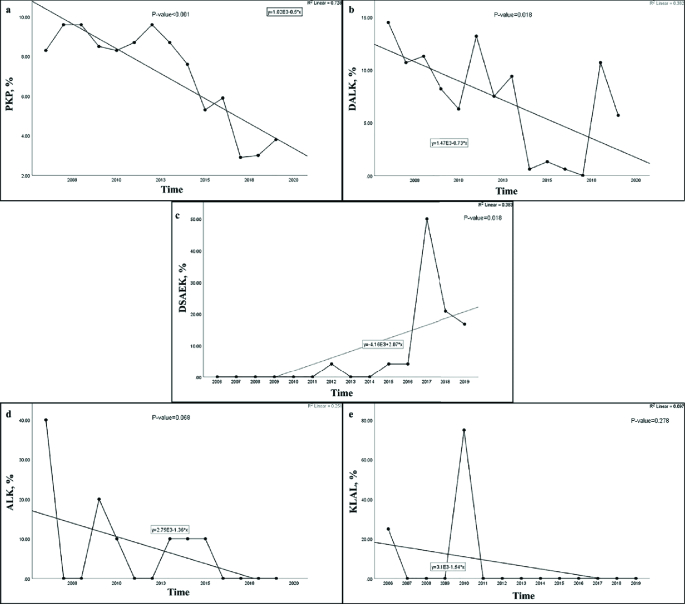
Trends of surgical techniques for pediatric keratoplasty in Iran between 2006 and 2019. Note the significant downward trend for PKP (a; *P* < 0.001) and DALK (b; *P* = 0.018); the remarkable ascending trend for DSAEK (c; *P* = 0.018); the borderline change of trend for the ALK (d; *P* = 0.068), and the lack of change of trend for KLAL (e; *P* = 0.278). PKP, penetrating keratoplasty; DALK, deep anterior lamellar keratoplasty; DSAEK, Descemet stripping automated endothelial keratoplasty; ALK, anterior lamellar keratoplasty; KLAL, keratolimbal allograft.

**Figure 4:**
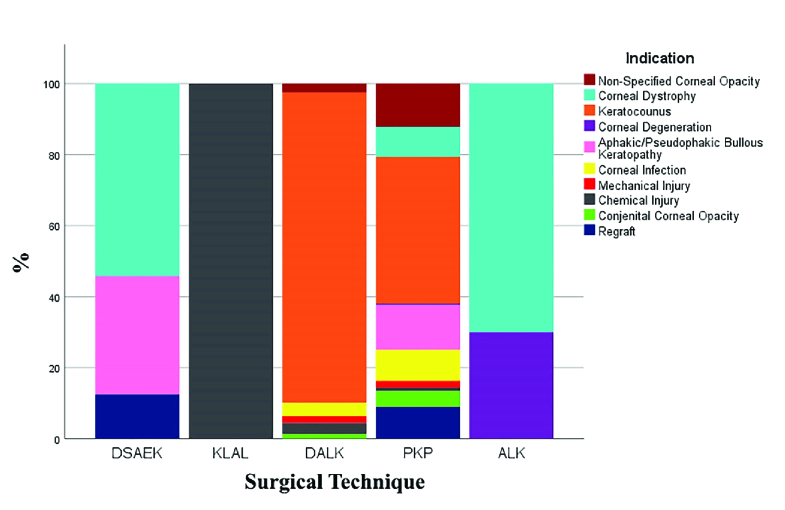
The proportion of indications for each surgical procedure. Keratoconus was the leading indication for PKP (41.3%) and DALK (87.4%), while corneal dystrophies were the main cause for DSAEK (54.2%) and ALK (70%). KLAL technique was exclusively used for chemical injuries (100%). PKP, penetrating keratoplasty; DALK, deep anterior lamellar keratoplasty; DSAEK, Descemet stripping automated endothelial keratoplasty; ALK, anterior lamellar keratoplasty; KLAL, keratolimbal allograft.

### Surgical techniques used for pediatric corneal transplantation

The most common surgical technique used for pediatric keratoplasty was PKP (1981, 90.9%) followed by DALK (159, 7.3%), DSAEK (24, 1.1%), ALK (10, 0.5%), and KLAL (4, 0.2%) [Table 2]. Although PKP remained the main type of keratoplasty performed over the 14 years, it showed a significant diminishing trend (*P* < 0.001) during the specified time period. Similarly, DALK showed a meaningful downward trend (*P* = 0.018) over the 14 years. Unlike the significant ascending trend in the rate of DSAEK (*P* = 0.018), the change of trend for ALK was borderline (*P* = 0.068), while for KLAL it was not significant (*P* = 0.278) [Figure 3].

In contrast to PKP, ALK, and KLAL which did not differ between the two age groups (*P* = 0.634, 0.082, and 0.178, respectively), DALK and DSAEK were more common in the older (*P* < 0.001) and younger (*P* = 0.002) age groups, respectively [Table 2].

The leading three indications for PKP were keratoconus (41.3%), aphakic/pseudophakic bullous keratopathies (12.6%), and non-specified corneal opacities (12.1%). The major leading indications for DALK, DSAEK, ALK, and KLAL were keratoconus (87.4%), CHED (54.2%), non-CHED corneal dystrophies (70%), and chemical burns (100%), correspondingly [Figure 4].

### Postoperative reports

Based on the postoperative data reported to the Central Eye Bank of Iran, except 69 (3.2%) eyes, the others (96.8%) reported graft clarity up to one month after corneal transplantation. In the category of short-term reports on postoperative unclear corneas, 50.7% were male, 53.6% aged > 12 years, and only three cases required regraft. The four primary indications for keratoplasty in this category were keratoconus (44, 63.8%), followed by non-specified corneal opacities (8, 11.6%), active corneal infections (6, 8.7%), and aphakic/pseudophakic bullous keratopathies (6, 8.7%). PKP (49, 71%) was the most common surgical procedure in this group, followed by DALK (19, 27.5%) and ALK (1, 1.4%).

##  DISCUSSION

The field of pediatric keratoplasty is relatively young and studies on surgical trends as well as indications for pediatric keratoplasty are limited. To the best of our knowledge, after the report of 2620 pediatric cases from the Eye Bank Association of America,^[14]^ the current survey is the largest case series reported for pediatric keratoplasty so far. The results of this cross-sectional study on the 14-year data for 2178 transplanted pediatric eyes, obtained from the Central Eye Bank of Iran, demonstrated that acquired nontraumatic corneal disorders, with keratoconus on the top of this category, were the major leading causes of pediatric keratoplasty and observed more commonly in the age group > 12 years. Even in the age group ≤ 12 years, keratoconus exceeded the congenital corneal abnormalities. PKP, in our survey, was the most common surgical technique for pediatric keratoplasty, similar to the other reports in which PKP was the only or the predominant surgical procedure for pediatric corneal transplantation.^[5,7,12,14,15,16,17,18,19,20]^

The indications for pediatric keratoplasty differ worldwide depending on the geographic region. For instance, in developed countries, congenital corneal disorders and nontraumatic acquired keratectasias are the leading indications for pediatric keratoplasty, while acquired corneal scars and ulcers of traumatic or infectious etiologies are on the top in developing countries.^[21]^ Table 3 summarizes published data on indications for pediatric keratoplasty in both developed and developing countries with the present study included.^[5,7,8,12,14,15,16,17,18,19,20,22,23,24,25,26,27,28,29]^ Keratoconus in our study, similar to the reports from pediatric keratoplasty in countries such as New Zealand, Italy, and Australia, was the most common indication for keratoplasty.^[14,15,16]^ This was in contrast to the reports from the United States, Singapore, Saudi Arabia, Eastern China, Malaysia, and India in which congenital corneal abnormalities and corneal infections were the top causes of keratoplasty in the pediatric age group.^[7,12,17,18,19,20]^ Congenital corneal abnormalities in our series ranked the second following acquired nontraumatic diseases; however, they were reported as the top indication for pediatric corneal transplantation in the United States (61.60%), Singapore (40.90%), Saudi Arabia (78.79%), and Eastern China (74.6%).^[12,18,19,20]^

The high prevalence of keratoconus in our keratoplasty series can be due to the common occurrence of vernal keratoconjunctivitis in Iran as well as challenges in wearing contact lens in the keratoconus pediatric patients, which had made keratoplasty the best option for this series of the patients.^[30]^ Nevertheless, the rate of keratoconus in our survey showed a descending trend over the 14-year period which can be due to the recent adoption of corneal cross-linking in such cases.^[30]^ Keratoconus in our series was also more prevalent in the elder age group, which may be explained by the surgeons' preference for performing keratoplasty in more advanced ages to achieve favorable surgical results.^[11,25,26]^

CHED, similar to the report from Saudi Arabia, was the most common corneal dystrophy in the pediatric age group in Iran.^[20]^ Zhu et al also reported congenital corneal abnormalities as one of the primary indications for corneal transplant in the youngest age group.^[27]^ While corneal dystrophies were the most common congenital corneal abnormalities in our series, they were not a common indication for pediatric keratoplasty in the other reports.^[8,24,27]^

As reported in Table 3, the rate of bullous keratopathies in our series (11.8%) was higher than the rate reported from Italy (6.1%), Eye Bank Association of America (2.3%), and New Zealand (1.7%).^[14,16,27]^ This may be explained by the increased adoption of cataract surgery for pediatric age group in Iran and the occurrence of corneal endothelial decompensation due to post-surgical complications. However, the number of pediatric keratoplasties due to aphakic/pseudopkakic bullous keratopathies did not show a change of trend over the 14-year period.

Non-specified corneal opacities ranked the third in the group of acquired nontraumatic diseases, and showed no significant change of trend during the 14 years. They were more common in the younger age group. As outlined in Table 3, the rate of corneal opacities of unknown etiologies was higher in the reports from developed countries such as USA (31.9%) and Singapore (20.5%) and from developing countries like India (19.1%) than the value reported in our survey (11.2%).[19, 26, 27] We assume that some of the non-specified corneal opacity cases in our series might have had infectious or traumatic etiologies which were missed by the corresponding surgeons when preparing the patients' data for the eye bank.

The rate of regraft in our survey (8.3%) was similar to the rates reported from developing and developed countries [Table 3]. There is only one report from the California, USA, in which regraft was the major leading indication for pediatric keratoplasty (43.4%).^[18]^ The regraft cases in our series, similar to the report by Zhao et al, was more common in the younger age group.^[12]^ Chronic endothelial graft rejection/dysfunction of the allograft was the predominant cause of graft failure in our series. A more active immune system has long been suggested as the main reason for the increased rate of corneal graft rejections observed in the younger recipients.^[31]^

Corneal infections were reported as the most common indication for pediatric keratoplasty not only in the developing countries such as India and Malaysia but also in a developed country such as Denmark (Table 3).[7, 17, 29] However, in our survey, it ranked the fourth in the category of acquired nontraumatic diseases and revealed a descending trend during the 14 years. This may be attributed to the public awareness on eye health and the presence of accessible eye care facilities for the treatment of ocular infections throughout the nation. Although corneal infections were accounted as the main cause of pediatric corneal graft failure in developing countries, only 5.3% (8 out of 151) of the active keratitis cases in our series were reported as graft failure following corneal transplantation.^[7]^

Acquired traumatic diseases in the literature accounted for 1.6% to 57.9% of pediatric keratoplasties [Table 3], ^[5][7][8][14,15,16][17][18][19][20][22][23][24][25][26][27][28][29]^ and were reported as the leading indication for pediatric corneal transplantation in both developed and developing countries.[8, 15, 24, 28] However, they were not common in the current study and accounted only for 3.2% of the transplanted eyes. Mechanical injuries were the most common cause in this category and were observed more frequently in the younger age group.

With the growing activity of the Central Eye Bank of Iran, there has been a growing trend in the annual rate of keratoplasty in Iran over the last decades; however, the annual rate of pediatric keratoplasty showed a significant diminishing trend over the 14-year period. This may be partially explained by the surgeons' preference for performing keratoplasty when the patients are over 18 years of age, especially in the keratoconus cases.

PKP was the most common surgical procedure for pediatric keratoplasty in our survey. In a study by Javadi et al,^[32]^ post-PKP graft quality in patients with CHED was relatively high up to three years after surgery. However, in the last decade, this technique of surgery has been replaced with the partial thickness procedures such as ALK in cases with anterior stromal involvements, DALK in keratoconus patients, and DSAEK in CHED, bullous keratopathies, and regraft. Given that PKP has a high risk of postoperative complications and graft failure, implementation of partial thickness procedures can be superior to the full thickness surgical techniques in certain pediatric corneal disorders.^[33,34,35]^ Nevertheless, the partial thickness procedures may have limitations such as the need for a committed pediatric cornea service, steep learning curve for the pediatric cornea surgeons, and unforeseeable visual outcomes.^[27,33]^ It was estimated that only about one-third of the currently practicing cornea surgeons are performing partial thickness procedures in pediatric cases.^[36]^

In our survey, the proportion of DSAEK showed a significant growth over the 14-year period and CHED was the main indication in half of the cases. This technique can be advantageous over PKP in pediatric cases with CHED due to the small corneal incision, use of a “closed system” condition, few sutures, and early suture removal causing rapid visual rehabilitation and improved visual outcomes.^[37]^ DALK is another partial thickness surgical procedure which was mainly performed for keratoconus eyes in our series. Although DALK has been reported with the same visual outcomes as PKP, while causing fewer complications and offering higher safety than the PKP, it had a significant descending trend over the 14 years in Iran.^[38,39]^ This may be mainly attributed to the downward trend of keratoconus as well as underreporting of some DALK cases to the eye bank by the corresponding surgeons.^[40]^

With the recent development in ocular surface reconstruction in Iran, keratolimbal techniques have been currently used for the patients with limbal stem cell deficiency.^[41,42]^ In our survey, KLAL was the main procedure for the management of chemical burn-induced ocular surface disorders in four pediatric cases. Implementation of this surgical procedure was not certainly specified in prior studies on pediatric keratoplasty.

The optical graft clarity was reported in the majority of the transplanted pediatric eyes (about 97%) in our series. Most of the eyes in the category of postoperative unclear corneas underwent PKP and the main indication was keratoconus.

The current study had limitations in terms of the retrospective nature of the survey and unavailable long-term follow-up data such as postoperative complications and final visual outcomes.

In summary, our survey was one of the largest case series reported on pediatric keratoplasty in terms of indications, surgical techniques, and postoperative optical clarity outcomes. In this survey, acquired non-traumatic corneal disorders were the most common indication for pediatric corneal transplantation in Iran, among which keratoconus was on the top. PKP was the most common surgical procedure, which, parallel to the decreasing rate of keratoconus, revealed a significant downward trend over the 14 years. Nevertheless, a part of the decline in the trend of PKP may be attributed to the shift from PKP to partial thickness procedures such as DSAEK for CHED and DALK for keratoconus.

##  Financial Support and Sponsorship

None.

##  Conflicts of Interest

The authors declare that they have no conflict of interest.
